# Precision Medicine and Melanoma: Multi-Omics Approaches to Monitoring the Immunotherapy Response

**DOI:** 10.3390/ijms22083837

**Published:** 2021-04-07

**Authors:** Fabio Valenti, Italia Falcone, Sara Ungania, Flora Desiderio, Patrizio Giacomini, Chiara Bazzichetto, Fabiana Conciatori, Enzo Gallo, Francesco Cognetti, Gennaro Ciliberto, Aldo Morrone, Antonino Guerrisi

**Affiliations:** 1 Oncogenomics and Epigenetics, IRCCS-Regina Elena National Cancer Institute, 00144 Rome, Italy; fabio.valenti@ifo.gov.it (F.V.); patrizio.giacomini@ifo.gov.it (P.G.); 2 Medical Oncology 1, IRCCS-Regina Elena National Cancer Institute, 00144 Rome, Italy; italia.falcone@ifo.gov.it (I.F.); chiara.bazzichetto@ifo.gov.it (C.B.); francesco.cognetti@ifo.gov.it (F.C.); fabiana.conciatori@ifo.gov.it (F.C.); 3 Medical Physics and Expert Systems Laboratory, Department of Research and Advanced Technologies, IRCCS-Regina Elena Institute, 00144 Rome, Italy; sara.ungania@ifo.gov.it; 4 Radiology and Diagnostic Imaging Unit, Department of Clinical and Dermatological Research, San Gallicano Dermatological Institute IRCCS, 00144 Rome, Italy; flora.desiderio@ifo.gov.it; 5 Pathology Unit, IRCCS-Regina Elena National Cancer Institute, 00144 Rome, Italy; enzo.gallo@ifo.gov.it; 6 Scientific Direction IRCSS-Regina Elena National Cancer Institute, 00144 Rome, Italy; gennaro.ciliberto@ifo.gov.it; 7 Scientific Direction, San Gallicano Dermatological Institute IRCCS, 00144 Rome, Italy; aldo.morrone@ifo.gov.it

**Keywords:** melanoma, immunotherapy, genomics, transcriptomics, proteomic, metabolomics, radiomics, liquid biopsy, precision medicine, biomarkers

## Abstract

The treatment and management of patients with metastatic melanoma have evolved considerably in the “era” of personalized medicine. Melanoma was one of the first solid tumors to benefit from immunotherapy; life expectancy for patients in advanced stage of disease has improved. However, many progresses have yet to be made considering the (still) high number of patients who do not respond to therapies or who suffer adverse events. In this scenario, precision medicine appears fundamental to direct the most appropriate treatment to the single patient and to guide towards treatment decisions. The recent multi-omics analyses (genomics, transcriptomics, proteomics, metabolomics, radiomics, etc.) and the technological evolution of data interpretation have allowed to identify and understand several processes underlying the biology of cancer; therefore, improving the tumor clinical management. Specifically, these approaches have identified new pharmacological targets and potential biomarkers used to predict the response or adverse events to treatments. In this review, we will analyze and describe the most important omics approaches, by evaluating the methodological aspects and progress in melanoma precision medicine.

## 1. Introduction

Melanoma is one of the more aggressive human tumors; representing 5% of all skin cancers. However, its great heterogeneity and ability to metastasize makes melanoma the skin tumor with the highest mortality rate [1]. During the early stages of disease development, melanoma has a favorable prognosis, and the 5-year survival rate affects almost all cases. Unfortunately, this condition drastically changes when the diagnosis is established at an advanced stage [2]. Only 15% of all melanoma metastatic patients survive three years after diagnosis [3]. Melanoma is constantly growing, from an epidemiological point of view, and is generally distributed in male patients between 25 and 50 years [4]. Melanocytes in the skin are the main site of melanoma development, but in a few cases, this pathology may originate from melanin-producing cells present in different mucosal surfaces, such as those of the gastrointestinal tract, the lining the choroidal layer of the eyes, and leptomeningeal [5].

Several studies have shed light on the mechanisms promoting melanoma development and have shown that the tumor transformation of melanocytes is complex and multi-stages. It is clear that most of the benign lesions present the alteration of v-Raf murine sarcoma viral oncogene homolog B (BRAF) in the codon V600E (sufficient for the nevus formation); but, for melanoma development, BRAF mutation is not sufficient because the disease progression is bound to concomitant alteration in other genes involved in the most important cellular processes [6,7]. Indeed, the benign nevi remain quiescent for several years and begin neoplastic transformation only after possible genetic mutations against target genes, such as telomerase reverse transcriptase (*TERT*), cyclin-dependent kinase inhibitor 2A (*CDKN2A*), phosphatase and tensin homolog deleted on chromosome 10 (*PTEN*), neurofibromin 1 *(NF1*), and v-kit Hardy-Zuckerman 4 feline sarcoma viral oncogene homolog (*KIT*). These genetic alterations are responsible for uncontrolled activation of mitogen-activated protein kinase (MAPK) and phosphoinositol-3-kinase (PI3K) pathways, physiologically involved in cell proliferation and survival [4].

Metastatic melanoma patient management has evolved considerably in recent years, by introducing “intelligent” treatments, such as targeted therapy and immunotherapy. In particular, immunotherapy, able to modulate and stimulate the activity of the patient’s immune system, represents a new frontier in the fight against cancer, and metastatic melanoma was one of the first solid tumors to benefit from these treatments. Indeed, melanoma is characterized, in its primary form, by the consistent presence of lymphocytic infiltrate [8]. Cytotoxic T-lymphocyte-associated protein (CTLA)-4 and programmed cell death protein (PD) 1/programmed death-ligand (PDL) 1-axis inhibitors have been approved by the Food and Drug Administration (FDA), in 2011 and 2014, respectively, and they have brought significant results in terms of survival [9]. Anti-PD1 treatments, in monotherapy, give a favorable response in 26–32% of cases, while the percentage increases to 60% when associated with ipilimumab (CTLA-4 inhibitor) [10,11]. Cancer cells benefit from uncontrolled activation of the CTLA-4 receptor and the PD1/PDL1 axis to block cytotoxic T-cell activity and evade immune surveillance [12]. In several tumor contexts, including melanoma, it is known that the interaction between PD1 and CTLA-4 and their ligands induces programmed death (apoptosis) of T lymphocytes by regulation of several pathways involved in cell survival and proliferation. Therefore, the ultimate goal of PD1 and CTLA4 inhibitors is to suppress this phenomenon [13,14,15]. The apoptotic process, physiologically important for cellular homeostasis, is profoundly altered in melanoma cells and not only. In them, indeed, the delicate balance between pro- and anti-apoptotic factors is largely in favor of molecules favoring cell growth [16]. Pro-survival molecules in the entire apoptotic pathway, which are often upregulated in cancer cells, include B-cell lymphoma-2 (BCL-2), B-cell lymphoma extra-large (BCL-XL), and myeloid leukemia cell differentiation (MCL-1) proteins. On the other hand, several pro-apoptotic proteins, such as caspases, are often downregulated in tumorigenic backgrounds [17]. In colon cancer, tumor cells induce apoptosis by activating PTEN phosphorylation and consequently inhibiting PI3K pathway. This implies, in T cells, the suppression of BCL-XL and induction of programmed death [13]. A study conducted in gastric adenocarcinoma further confirmed this phenomenon. Indeed, in a cohort of 60 patients, the expression of PD1 and PDL1 in peripheral blood and tumor-infiltrating cells was characterized and the association between their expression and disease progression was evaluated. The authors observed that PD1 expression in the bloodstream and on T cells increased with disease progression and, in vitro, the lymphocytes induced PDL1 expression on tumor cells by promoting their apoptosis. Inhibition of PDL1 has reversed this effect [14]. It is clear that the efficacy of immunotherapy could be increased by combining these treatments with specific molecules that control PDL1 expression and cell proliferation. For example, it is known that several transcription factors, such as hypoxia-inducible factor-1α (HIF-1α) and signal transducer and activation of transcription-3 (STAT3), and several microRNAs (miR), such as miR-570, miR513, miR-197, miR-34a, and miR-200 act by regulating PDL1 [18].

As observed for other therapeutic approaches, a high percentage of patients do not respond to immunotherapy or suffer from adverse events. Based on these observations and on the many evidences that the tumors are complex structures, there is a necessity to invest in precision medicine. In this scenario, the omic sciences are applied; they allow an extensive analysis of all tumor characteristics and support the clinical management of the melanoma patient.

In this review we will illustrate the most important omics applications in melanoma prediction of positive or negative effects to immunotherapy.

## 2. Precision Medicine in Melanoma

Oncological research has significantly improved the clinical evolution of many cancers considered incurable, such as metastatic melanoma. To date, precision medicine allows predicting responses to treatments or possible adverse events through the discovery and analysis of new predictive and/or prognostic biomarkers, reducing the gap between basic research and clinical management of the patient.

**Figure 1:**
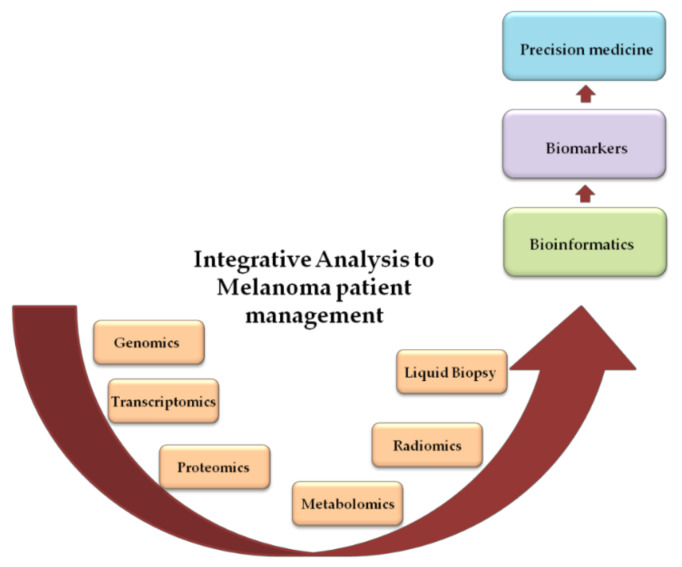
Schematic representation of data flow from basic to clinic research.

In Figure 1, the principal omics analyses related to melanoma are summarized.

### 2.1. Genomics Approaches

It is well known that cancer is a disease with a high genetic component. Indeed, several mutations in genes involved in the most important cellular processes responsible of tumorigenesis have been identified [19,20]. Genomic analysis, i.e., the study of genome functions through sequencing, currently make it possible to identify DNA mutations, rearrangements, or amplifications in the single patient and direct them towards specific treatments [21,22]. The development of next-generation-sequencing (NGS) techniques has made it possible to sequence tumor DNA in a short time and a low cost. The detection of driver and germ mutations, and the quantification of the tumor mutational burden (TMB), have enhanced, in the clinical setting, the approach to precision medicine [23].

Melanoma development and its great invasion capability are essentially due to many somatic mutations, which alter the physiological function of two molecular pathways: MAPK and PI3K/protein kinase B (AKT) pathways. Both these pathways are involved in signal transduction from the plasma membrane to the nucleus by the activation of several proteins often mutated, not only in melanoma, but in many other type of cancers [24,25]. The MAPK pathway represents the most dysregulated site in melanoma and is fundamental for the uncontrolled cell proliferation and differentiation; about 50–60% of all melanomas are characterized by a somatic mutation of serine/threonine protein kinase BRAF, in the codon V600E [26]. Instead, the MAPK signaling is aberrant activated also by missense mutation charged to neuroblastoma RAS viral oncogene homolog (NRAS), a member of the RAS gene family, involved in the regulation of cell growth, and particularly important for melanoma onset [27]. NRAS is mutated in 15–20% of all melanomas and is correlated with a more aggressive disease subtype with elevated invasive capabilities [28]. In melanoma, although present in much lower percentages compared to BRAF and NRAS mutations, in the last few years, several mitogen-activated protein kinase kinase (MEK) mutations have been identified. In a sequencing study conducted in 2011, Nikolaev and his collaborators have individuate in about 8% of the samples analyzed, mutations of MEK1 and MEK2 involved in constitutive Extracellular Signal-Regulated Kinase (ERK) activation [29]. Stark M.S. et al. have identified somatic inactivating mutations of mitogen-activated protein kinase (MAP3K) 5 and 9, which lead to a reduction in kinase activity and are associated to chemoresistance [30]. Moreover, somatic mutations of MAP3K5 are responsible for lower pro-apoptotic capacity and, consequently, they determine an increase in cell proliferation [31].

PI3K pathway, although less frequently than the MAPK signaling, is characterized by uncontrolled activity in melanoma [32,33]. PTEN is an important tumor suppressor gene involved in cell growth, survival, and cell motility. Through its protein phosphatase activity, it is able to dephosphorylate the phosphatidylinositol (3,4,5)-trisphosphate (PIP3) in phosphatidylinositol (4,5)-bisphosphate (PIP_2_), and this function makes PTEN an important negative regulator of PI3K pathway. In melanoma, PTEN is often mutated/deleted, and its loss of function is present and concomitant with BRAF mutations in about 44% of melanomas [34]. In this context, several studies have shown that PTEN loss activity promotes melanoma development by upregulation of the PI3K pathway, with consequent promotion of cell survival and apoptosis reduction [35,36]. AKT is a downstream effector of the PI3K signaling and is involved in the phosphorylation and inactivation of many proteins. Three isoforms of this protein have been identified (AKT1, AKT2, and AKT3), often mutated in different cancer types. In 2008, Davies and his collaborators, analyzing a large panel of melanoma tissues and cells, have occasionally observed mutations of AKT1 and AKT3, but the contribution of these mutations in the melanocyte transformation process remains unclear [37].

Other mutations, with a lower frequency as compared to those described above, have been identified in melanoma. Activating somatic mutations in the KIT gene were found in approximately 2–8% of all melanomas, even though they are particularly associated with mucosal and acralic forms of this pathology (10–20%, respectively) [38,39]. These mutations are generally not expressed simultaneously with the more frequent BRAF and NRAS [24,40]. NF1 encodes for a cytoplasmic protein, which regulates and inhibits the RAS activity through hydrolysis of GTP in GDP. This protein, considered an important tumor suppressor, is mutated in about 15% of all melanomas and represents the third gene most altered in this pathology. Most of the NF1 mutations are characterized by a loss of function, leading to NRAS iper-activation and, consequently, MAPK and PI3K pathways dysregulation. Loss of NF1 is associated by a perennial activation of MAPK signaling and determines resistance to RAF and MEK inhibitors [41,42]. Telomerase represents a complex machinery used by the cells for the maintenance of the normal telomere homeostasis. It is formed by a catalytic subunit *TERT* and a telomerase RNA component (TERC). *TERT* promoter mutations result in several cancer types, including melanoma, and the simultaneous presence with *BRAF/NRAS* alterations is associated with poor prognosis for the patients. These mutations, by stabilizing short telomeres, determine genomic instability, and uncontrolled cell proliferation, and are associated with MAPK activation [43,44].

The clinical outcome of immunotherapy is not easy to predict and, in some cases, is not positive for patients. Indeed, unlike targeted therapy, which has predictive response markers, such as the BRAF mutation, validated biomarkers for the immunotherapeutic agents still need to be approved [45,46]. In recent years, gene sequencing studies have shown that the TMB, neoantigen load (NL) or PDL1 expression degree are often associated with an increased response to immunotherapy [47,48]. However, undefined cut-off and weak reliability of prediction have not given significant results [46,49]. Thus, in melanoma, the research of new predictive biomarkers appears fundamental to discriminate the patients and to avoid unnecessary and sometimes dangerous treatments. In this context, a very recent work has generated a genetic mutation model, called immunotherapy score (ITS), with a high ability to predict the response to immunotherapeutic agents. The authors have produced this model by sequencing whole exomes, and have observed an increased response to treatments and prolonged survival in patients, with high levels of ITS. ITS is not only a good isolated predictive factor, indeed, in association with TMB and plasmatic value of lactate dehydrogenase (LDH), it seems to be, in terms of response to immunotherapy, the best biomarker compared to single factors [46]. A new possible marker of response to immunotherapy in metastatic melanoma is the activation of nuclear factor kappa-light-chain-enhancer of activated B cells (NF-kB) signaling. Carol Amato and collaborators, by pre-treatment sequencing of DNA and RNA of melanoma patients, have found a higher mutational load of NFKBIE (NF-kB negative regulator), in codons G34 and G41, only in patients more responsive to anti-PD1 therapy. NFKBIE loss of function resulted in the activation of the NF-kB pathway, which, therefore, can be considered a possible predictive factor of treatment response [50]. Moreover, alterations to DNA damage repair (DDR) pathways are associated with a better response to immune checkpoint inhibitors (ICIs). Hugo and his research group have observed, in a cohort of metastatic melanoma patients responding to PD1 blockade, an increase of mutations in the homologous recombination (HR) repair gene BRCA2 [51].

As previously mentioned, melanoma is characterized by a complex and heterogeneous structure, and this condition is associated with a major presence of tumor subclones, able to bypass the immune system and drug blocking. For these reasons, tumor heterogeneity is considered another parameter of response to ICIs. Several studies have shown that patients with less heterogeneous melanoma responded better to the blocking action of anti-CTLA-4 and anti-PD1 [52,53]. In addition to the various factors already described, a study conducted on 144 patients with metastatic melanoma included purity and ploidy of the tumor as predictive markers of response to PD1 inhibitors. Specifically, higher tumor purity was associated with tumor progression, while the ploidy was lower in non-responding patients [54].

### 2.2. Transcriptomics Approaches

Transcriptomics represents the study and analysis of the entire transcriptome, i.e., all cellular mRNAs, precursors of proteins. mRNA is the product of gene expression, its evaluation is fundamental to understand the functionality of DNA in a particular context. Currently, transcriptomics is a widespread molecular approach and the methods associated with it are: (1) microarrays, which quantify only a selected set of sequences; (2) fast RNA sequencing (RNA-Seq), which captures all sequences [55].

The need to early discriminate patients, responding or not responding to immunotherapy, has led to the construction of predictive signature, such as IMmuno-PREdictive Score (IMPRES). It is a predictor response to ICIs, which includes 15 pairwise transcriptomics relations between 28 immune checkpoint genes [56]. Unfortunately, the high melanoma heterogeneity often neutralizes or minimizes the prediction ability of these molecular signatures. Therefore, the attention has shifted from the melanoma to its microenvironment and a recent and interesting study has analyzed 94 melanoma samples collected at baseline and at progression, after treatment with anti-PD1. Using RNA-seq analysis, the authors observed that the downregulation of major histocompatibility complex (MHC) class I is associated with resistance to PD1 inhibitors and a de-differentiated MITFlow/AXLhigh phenotype. This condition is modulated by transforming growth factor-β (TGF-β) and, for this reason, combinations of anti-TGF-β and PD-1 inhibitors could provide important therapeutic benefits [57].

### 2.3. Proteomics Approaches

The integrated approach of multiple disciplines allows for an extensive view, especially in highly heterogeneous tumor contexts, such as melanoma. Indeed, genomic and transcriptomic studies alone highlight only a slice of its complexity. The several post-transcriptional modifications do not allow a linear relationship between gene expression and final product and the proteins obtained often differ from the starting gene. For these reasons, in-depth analysis of proteins are essential to obtain more detailed information about the tumor [58]. Proteomic studies can be performed either on cell- and tissue-derived source or on specific samples, such as blood. This occurs especially when the material to be analyzed is limited, as in the case of metastatic melanoma [59].

Variations in protein expression or function could determine response or non-response to treatments, such as immunotherapy. Recently, 116 stage IV melanoma patients undergoing tumor-infiltrating lymphocytes (TILs) or anti-PD therapies were involved for proteomics analyses. Both groups of patients were divided into responders and non-responders and, by high-resolution mass spectrometry, their protein composition has been analyzed. The authors have observed significant changes in lipid and oxidative metabolism between patients who responded to both treatments and those not responding. Specifically, the lipid and ketone metabolisms are the worst mediators of tumor immunogenicity and of antigen presentation by cancer cells [60].

The plasma proteome is profoundly dynamic and fluctuates under the influence of several factors, such as drug treatments. The use of high-resolution isoelectric focusing liquid chromatography-mass spectrometry (HiRIEF LC–MS/MS) and antibody-based targeted proteomics with proximity extension assays (PEAs) has allowed to analyze the protein structure of blood samples from metastatic melanoma patients treated with immunotherapy and to identify plasma biomarkers [61]. For this aim, the authors of the study have used the pre- and post-treatment plasma with ICIs of 46 melanoma patients (stage IV) and have compared it with the samples of patients undergoing target therapy. The most striking result of this screening was represented by the increase in circulating levels of PD1 only in response to anti-PD1 treatment, and in patients responding to this therapy, compared to the control group with targeted therapy. The plasmatic PD1 increase provides endogenous PDL1 inhibition in parallel with therapy-induced inhibition [61].

Although immunotherapies have made important advances in terms of survival, they are not free of adverse events that preclude treatment outcomes and, even, patient survival. One of the targets of proteomics analysis is also to identify possible biomarkers involved in toxicity and adverse events to treatment. In metastatic melanoma, specific proinflammatory cytokines, such as interleukin (IL)1a, IL2 and interferon (IFN) α2, could help in the early management of severe immunocorrelated toxicity. Indeed, a study conducted in melanoma patients treated with ICIs has found significant upregulation of 11 cytokines in the cohort with severe immune-related toxicities at baseline (PRE) and early during treatment (EDT) compared to the control group [62].

### 2.4. Metabolomics Approaches

The study of the metabolome, i.e., the low molecular weight products of the cellular processes, is fundamental to understand the functional status of cells. Together with genomics, transcriptomics, and proteomics, it provides useful information about the state of cellular “health” and the continuous interactions with the microenvironment [63]. Cancer cells metabolism is profoundly altered and, therefore, produces molecules that are specific and typical of non-physiological conditions. Metabolomics approaches can be targeted, if they focus on detecting a few and specific metabolites, or non-targeted if they attempt to identify many metabolites from biological fluids as possible [64]. Several plasma biomarkers, indicators of metastatic capacity, tumor progression, and drug response, have been identified for melanoma: however, being common to other diseases or, in general, to inflammatory processes, they are not considered reliable markers.

Among all serum biomarkers, LDH is certainly the most specific for melanoma and, in association with other predictive and prognostic factors, it is often used to predict drug responses. It is involved in the conversion of pyruvate to lactate and is upregulated in melanoma due to increased cell necrosis that spills the enzyme into the bloodstream. In patients with advanced melanoma, elevated LDH levels are associated with poor prognosis and are directly related to survival with a specificity of 92% [65]. Over the years, several studies have evaluated the prognostic role of LDH in response to immunotherapy with conflicting results. Some studies have evaluated that high baseline LDH levels correspond to reduced survival for advanced melanoma patients and decreased response to ICIs [66,67]. For this reason, new possible drug combinations have been proposed to overcome resistance to immunotherapy. High LDH levels are the consequence of increased glycolytic activity of the tumor in response to hypoxia. Therefore, the combinations of ICIs with glycolysis inhibitors or vascular endothelial growth factor (VEGF) inhibitors could open the way to new therapeutic scenarios [67]. On the other hand, a recent study re-evaluated the results of the KEYNOTE-001 clinical trial. Indeed, the authors observed a greater and more durable response to pembrolizumab in advanced melanoma patients with elevated baseline LDH levels compared to those with normal enzyme levels [68]. In some cases, BRAF-mutant melanoma patients after a first line of treatment with targeted therapy receive subsequent treatment with ICIs. The response to immunotherapy was significantly better in patients with normalized LDH levels after targeted therapy, compared to those with still high levels of the enzyme [69]. This result suggests that the assessment of serum LDH levels, after treatment with targeted therapy, could be a valid index of response or non-response to subsequent immunotherapy.

Albeit, only LDH is recognized in the Joint American Commission on Cancer (AJCC) melanoma guideline, protein S100 is another biomarker used for this pathology. Elevated S100B levels are an indicator of metastasis, response to treatment, disease relapse, and overall survival (OS) [70,71]. Recently, basal S100B levels have been considered a valuable guide to therapeutic choices in patients with metastatic melanoma undergoing immunotherapy. Indeed, it has been observed that the patients treated with pembrolizumab, in monotherapy, or in combination with ipilimumab, and with simultaneous elevated baseline levels of S100B and LDH, showed significantly reduced OS, compared to the patients with normal S100B [72].

### 2.5. Radiomics Approaches

Radiomics is a promising, multi-step emerging approach that might be of support to precision medicine by extracting quantitative, tumor-specific features from radiological biomedical images. In the era of machine-learning and artificial intelligence, the radiomic information obtained from medical images can provide quantitative objective parameters and play a crucial role in clinical decision support and cancer management [73]. Indeed, the association between radiomics features and the clinicopathological information of diseases could help to optimize the treatment selection and management of cancer patients [74].

Main radiomics steps are: acquisition of biomedical images, extraction of a wide number of quantitative imaging-based features, and correlation of these with different endpoints [75]. More in detail, high-quality, standardized images were firstly acquired with modern Computed tomography (CT), Magnetic Resonance Imaging (MRI), or more rarely with combined Positron Emission Tomography (PET)/CT scans. Then, the lesions are delineated manually or using automated techniques (“segmentation”); successively quantitative parameters related to texture, shape, and intensity of the lesions are extracted using high-throughput methods, and both two-dimensional (2D) and three-dimensional (3D) features can be obtained. Finally, correlations were investigated between extracted features and the specific clinical endpoints, such as treatment response or overall survival. Texture analysis (TA) is one of the most widely spread radiomics methods able to describe the textural properties of the images by analyzing its grey-level patterns and quantitative histogram [76]. TA is not invasive and able to provide quantitative and spatial information over the volume of lesion and organs at multiple time points [75]. Thus, TA might represent an added value to the analysis of tissue samples that are generally performed only once in a specific tumor site [77]. For this reason, there has been an explosive rise in popularity of radiomic/TA topics and still even more literature continue to be produced, resulting in many methods and applications. In particular, almost all the papers published on the last decade investigated cohorts of patients that underwent MRI or CT, while only a few part of them involved different imaging modalities such as PET or PET/CT [77]. Indeed, clinical trials on melanoma generally involve as standard follow-up protocol CT scan with Response Evaluation Criteria in Solid Tumours (RECIST) or MRI when higher resolution is needed as for brain studies [78]. The software mostly used for radiomics/TA are PyRadiomics Python package, TexRAD software, the IntelliSpace Portal V.8 Philips Healthcare, and LifeX software, a MATLAB toolbox Radiomics implemented by Vallieres et al. [79]. Sometimes, the radiomics/TA was made with in-house built software as reported in [80,81].

As above-mentioned, the last step of a radiomics/TA procedure is to construct mathematical models or classifier that can provide prognostic information or be predictors of treatment response. A classifier built using the radiophenotypical properties of the tumor could help the patients stratification and to optimize clinical decision [77]. A wide range of methods for classifier development and validation are reported in literature. For example, Trebeschi et al. have demonstrated that radiomics can automatically extract specific biomarkers to predict immunotherapy response using machine learning techniques to build a radiomic-based classifier [82]. Smith et al. have validated the use of CT-based radiomic to predict immunotherapy response, reporting that a decrease in a radiomic feature (mean positive pixel or MPP) was present on initial posttherapy CT for patients with a greater risk of mortality [83].

The role of radiomic/TA in predicting the response to immunotherapy in metastatic melanoma patients treated with pembrolizumab has been investigated by Durot et al. [84]. The authors have found the radiomic feature named skewness to be a potential predictor of outcome. In a recent retrospective study by Schraag et al., the authors have revealed another feature, named kurtosis, as an independent predictor of overall survival in melanoma patients treated with immunotherapy [85].

In general, radiomic/TA can provide novel imaging-based biomarkers to be correlated with a panel of diagnostic, prognostic, and predictive biomarkers, either serological (i.e., LDH), molecular (i.e., TMB), and immunohistochemical (i.e., PD-L1), to support the management of advanced melanoma patients [49,86]. Moreover, a major strength of radiomics is that information are extracted directly from biomedical imaging that are routinely obtained for almost every oncological patient, with no need of further acquisitions or costs. Despite the promising clinical advantages of radiomics, there are some issues that must be accurately evaluated for validating the prognostic role of imaging-based radiomic features. An appropriate methodological approach is needed in order to select robust methods and to provide reproducible data [73]. Moreover, multicentric studies with larger cohorts are strictly recommended to validate radiomic features as biomarkers on a wide scale.

## 3. New Frontiers in Precision Medicine: Liquid Biopsy

Although not yet clinically recognized, liquid biopsy is rapidly assuming a key role in the search for specific biomarkers for various cancer forms, such as non-metastatic colorectal cancer and melanoma [87,88,89]. Conventional biopsy techniques often produce limited information about tumor status, because they capture only a minor part of the tumor mass at a specific time. However, as it has been known, the tumor and its microenvironment are constantly evolving, and can change in response to treatments. In this scenario, liquid biopsy finds fertile ground and is considered a new prognostic and predictive technique to treatment response. This non-invasive technique essentially allows the isolation and detection of circulating tumor cells (CTCs), circulating tumor DNA (ctDNA) and exosomes, often released in the peripheral blood by cancer cells [90].

### 3.1. CTCs

CTCs, released by primary tumor or metastasis, are present in peripheral blood. The transition to a mesenchymal phenotype, mediated by epithelial mesenchymal transition (EMT) process, indeed, allows tumor cells to acquire invasive and migratory properties and to penetrate in the blood and lymphatic circulations. This prerogative induces the recruitment of CTCs in sites distant from the primary tumor to form metastases. In addition, these cells can interact with elements of the stroma and immune system to evade the immune response [91,92]. The identification and quantification of CTCs has, potentially, an important prognostic value because it would be related to the response to treatments; the limited number of CTCs in the blood (1–10 cells per milliliter of whole blood) has made necessary to develop more sensitive methods for their isolation [93]. In recent years, several experimental methods have been proposed to standardize the isolation of CTCs, based essentially on their physical and biological characteristics [94,95]. CellSearch^®^ system (Menarini Silicon Biosystems, Inc.) is the only assay approved and standardized, in 2004, by the FDA, for the detection of CTCs in several solid tumors, such as breast, colon, and prostate cancer [96,97,98]. The assay discriminates tumor cells in the blood based on their expression of epithelial cell adhesion molecule (EpCAM) [99]. CellSearch^®^ kits have also been developed for melanoma, but there are still too few studies conducted [100]. In addition, melanoma CTCs poorly express EpCAM and, therefore, new potential markers are being examined to discriminate them [101]. Recently, the new EPISPOT assay (S100-EPISPOT assay) has been designed for melanoma CTCs; this assay discriminates the cancer cells in the blood based on their expression and secretion of the protein S-100, typical of melanoma. The study has shown that the sensitivity of S100-EPISPOT was significantly higher than that of CellSearch [102].

In many cancer contexts, the detection of CTCs can provide important diagnostic information and is related to patients’ outcomes. In a recent study, a dual-step procedure of CTCs separation was evaluated in blood samples of 17 patients with advanced melanoma. The authors, observing parameters, such as progression free survival (PFS), OS, and number of metastasis sites, have highlighted that CTCs’ amount is correlated with patient prognosis. However, being based on a limited number of samples, these results will need to be further confirmed [103]. A clinical study has evaluated the association between the presence of CTCs in stage III melanoma patients and disease relapses. The analysis was conducted on 243 patients at first clinical presentation and the detection of CTCs was significantly associated with a shorter relapse-free survival (RFS), 6 months in 37% of patients enrolled [104].

Currently, 11 melanoma clinical trials involve the use of CTCs (Table 1) [78].

Melanoma CTCs appears to be a predictive biomarker to immunotherapy response. A significant correlation has been observed between CTCs, with elevated basal levels of PDL1, and pembrolizumab response in advanced melanoma patients. Patients with PDL1+ CTCs have found favorable effects in terms of PFS as compared to the control group with low levels of circulating PDL1 [105]. The development of a signature of 19 genes for melanoma CTCs (CTC score) allowed to quickly assess the response to ICIs. CTCs score changes radically after treatment with immunotherapy, providing important information on the treatment response even in the long term. Therefore, early monitoring of CTCs changes could help clinicians in screening patients for immunotherapy and it would also avoid unnecessary and harmful treatments for non-responsive patients [106].

### 3.2. ctDNA

ctDNAs are small fragments of nucleic acid, released by CTCs through unclear mechanisms. These DNA fragments may likely be associated with necrosis or apoptosis phenomena [107,108]. On the other hand, the release of ctDNA in the bloodstream can also actively occur from live cells, and this method allows the establishment of a genomic instability typical of the metastatic process [109,110]. The first scientific evidence that correlated ctDNA to the presence of a tumor was found in 1977. Higher concentration of ctDNA in the blood was detected in patients with pancreatic cancer; in addition, a significant decrease of its levels was observed after pharmacological treatment [111]. Although ctDNA and CTCs are considered important biomarkers and are present in many advanced tumors, to date, few studies have analyzed them in the same patients [107]. The blood concentration of ctDNA in cancer patients is only a small fraction of the total amount of DNA released even by “normal” cells. To date, two experimental methods are mostly used for the isolation of ctDNA: (1) recognition and detection of specific mutations that characterized the neoplasm of interest; (2) detection of new mutations or somatic variations in ctDNA [112]. The hypothesis that the DNA released from cancer cells is biologically active has been confirmed in a very interesting preclinical study. The authors have observed that ctDNA stimulates cellular transformation and tumorigenic process. Indeed, DNA-depleted supernatant of cells and colon cancer patients blocks malignant transformation of NIH3T3 murine cells [113].

Early assessment of ctDNA change during therapy could help clinicians to predict tumor response or non-response to immunotherapy [114,115,116]. Some immunotherapy methods do not provide immediate benefit to the patient, but require a longer time period. For example, in the case of autologous transfer of TILs, the complete response may occur even after 1–2 years. Therefore, clinicians are often confronted with uncertain outcomes and need to find biomarkers that can provide early information. Changes in BRAF V600E ctDNA levels, within the first month after T-cell transfer immunotherapy, can be used to rapidly identify responding from non-responding patients. Analysis of blood samples of 48 metastatic melanoma patients has showed a tight correlation between the development of an early BRAF V600E ctDNA peak and complete response to treatment with TILs [117]. A recent study demonstrated that pre-treatment ctDNA levels can be used to stratify patients to undergo to first-line of ICIs. Indeed, melanoma patients with lower pre-treatment plasma ctDNA levels had a longer PFS. On the other hand, patients with higher ctDNA values showed less inauspicious outcomes only when treated with anti PD1/CTLA-4 combinations and not with anti-PD1 alone [118]. These findings were partially confirmed by an additional clinical trial performed in 85 patients with metastatic melanoma and undergoing anti-PD1 treatment. Analysis of liquid biopsies, obtained before and during treatment, has revealed that patients with undetectable ctDNA before therapy have responded significantly better than those with higher ctDNA values (median PFS 26 weeks versus 9 weeks, *p* = 0.01) [119].

### 3.3. Exosomes

Exosomes are vesicles surrounded by plasma membrane and released by cells into microenvironment. They may contain proteins, lipids, or genetic material, and are often used for intercellular communication [120]. In recent years, exosomes have been the focus of study in oncology because also cancer cells produce and release these vesicles and they could be involved in the many stages of tumor progression and drug resistance [121,122]. In addition, given their abundance in circulation, exosomes are considered as potential biomarkers for cancer treatment [123].

Tumor cells implement several mechanisms to evade the immune system control. One of these involves the release of exosomes that carry PDL1, still bound to the plasma membrane, into microenvironment. Consequently, the tumor enhances its immune evasive potential and develops resistance to ICIs treatments [122]. Exosomal PDL1 can either antagonize anti-PD-L1 therapy by binding to the antibody itself or, being resistant to anti-PD-1 therapy, can suppress T-cell activity directly and/or indirectly [124].

## 4. Future Perspectives

The next few decades will focus on the development of increasingly targeted and precise medicine. The study of specific tumor forms, indeed, has highlighted the high heterogeneity among patients, and treatments useful for some patients may not be considered equally valid for others. Melanoma represents one of these tumor contexts that, influenced by multiple immunological factors and a pro-cancerous microenvironment, evade pharmacological treatments in a form still not completely clear. Therefore, it is important to evaluate all molecular and diagnostic aspects in order to have an overall view before subjecting a patient to possibly unnecessary treatments. Therefore, the omics sciences are the “future” on which to invest, appropriately, not only at the pre-clinical level (as already happens for melanoma and not only), but also at the clinical level to customize the intervention of the oncologist. Multidisciplinary approaches involving different professional figures (clinicians, biologists, mathematicians, informatics, and so on) appear to be fundamental, and aim to brilliantly overcome errors that today are insoluble with standard medicine. For melanoma, radiomics and liquid biopsy represent the new approaches on which to invest and focus. Compared to all the methods described, indeed, they are not yet used in the clinical setting for the identification of new biomarkers, or to early assess the response, or not, to a particular treatment. However, given their obvious potential, as a corollary of other more defined and used approaches, they can certainly bring benefits to the treatment of the patient.

## 5. Conclusions

The clinical evaluation of the cancer patients has undergone profound changes in recent decades. The tumor is no longer considered a separate entity, but is in constant dialogue with its surrounding microenvironment. Moreover, melanoma is a highly heterogeneous tumor form and differs profoundly between patients. Therefore, precision medicine has paved the way for increasingly personalized treatments, seeking to minimize the proportion of non-responding patients. Today, the dialogue between the various experimental approaches and increasingly precise data evaluation techniques allow to have a complete view of the patient, avoiding harmful and controversial treatments.

## Figures and Tables

**Table 1:** CTCs involvement in melanoma clinical trials.

ID Number	Status	Study Type	Outcome Measure
**NCT01528774**	Completed	Observational	CTCs isolation and DNA mutation analysis
**NCT01573494**	Completed	Interventional	CTCs isolation and evaluation in metastatic melanoma patients, before and after treatment. Contribution of CTCs in patient’s survival.
**NCT01558349**	Completed	Observational	Comparing the EPISPOT and CellSearch Techniques for CTCs isolation.
**NCT01776905**	Recruiting	Observational	Evaluation of photoacoustic flow cytometry (PAFC)-based prototype for CTCs isolation.
**NCT03797053**	Unknown	Observational	Evaluation of CTCs as predictive biomarkers in treatment response
**NCT01565837**	Unknown	Interventional	Evaluation of CTCs as predictive biomarkers in treatment response
**NCT02862743**	Active, not recruiting	Interventional	Molecular characterization of advanced melanoma
**NCT00338377**	Recruiting	Interventional	CTCs analysis and patient’s survival evaluation
**NCT02071940**	Unknown	Interventional	CTCs analysis and evaluation of response to treatment
**NCT03007823**	Completed	Interventional	CTCs analysis and patient’s survival evaluation
**NCT01878396**	Unknown	Observational	Evaluation of CTCs as predictive biomarkers in treatment response

CTCs: circulating tumor cells; photoacoustic flow cytometry (PAFC).

## Abbreviations

The following abbreviations are used in this manuscript.

2D	Two-dimensional
3D	Three-dimensional
AJCC	American Joint Commission on Cancer
AKT	Protein kinase B
BCL-2	B-cell lymphoma-2
BCL-XL	B-cell lymphoma-extra large protein
BRAF	v-Raf murine sarcoma viral oncogene homolog B
CDKN2A	Cyclin-dependent kinase inhibitor 2A
CTCs	Circulating tumor cells
ctDNA	Circulating tumor DNA
CTLA-4	T-lymphocyte-associated protein
CT	Computed tomography
DDR	DNA damage repair
EMT	Epithelial mesenchymal transition
EpCAM	Epithelial cell adhesion molecule
ERK	Extracellular Signal-Regulated Kinase
FDA	Food and Drug Administration
HIF-1α	Hypoxia-inducible factor-1α
HiRIEF LC-MS/MS	High-resolution isoelectric focusing liquid chromatography-mass spectrometry
HR	Homologous recombination
ICI	Immune checkpoint inhibitor
IFN	Interferon
IL	Interleukin
IMPRES	IMmuno-PREdictive Score
ITS	Immunotherapy score
KIT	v-kit Hardy–Zuckerman 4 feline sarcoma viral oncogene homolog
LDH	lactate dehydrogenase
MAP3K	Mitogen-activated protein kinase kinase
MAPK	Mitogen-activated protein kinase
MCL-1	Myeloid leukemia cell differentiation
MEK	Mitogen-activated protein kinase kinase
MHC	Major histocompatibility complex
miR	microRNA
MRI	Magnetic Resonance Imaging
NF1	Neurofibromin 1
NFκB	Nuclear factor kappa-light-chain-enhancer of activated B cells
NGS	Next-generation-sequencing
NL	Neoantigen load
NRAS	Neuroblastoma RAS viral oncogene homolog
OS	Overall Survival
PD1	Programmed cell death protein 1
PDL1	Programmed death-ligand 1
PEAs	Proximity extension assays
PFS	Progression Free Survival
PI3K	Phosphoinositol-3-kinase
PIP2	Phosphatidylinositol (4,5)-bisphosphate
PIP3	Phosphatidylinositol (3,4,5)-trisphosphate
PET	Positron Emission Tomography
PTEN	Phosphatase and tensin homolog deleted on chromosome 10
RECIST	Response Evaluation Criteria in Solid Tumours
RFS	Shorter relapse-free survival
RNA-seq	Fast RNA sequencing
STAT3	Signal transducer and activation of transcription-3
TA	Texture analysis
TERC	Telomerase RNA component
TERT	Telomerase reverse transcriptase
TGF-β	Transforming Growth Factor-β
TILs	Tumor-infiltrating lymphocytes
TMB	Tumor mutational burden
VEGF	Vascular endothelial growth factor
